# Non-native earthworms increase the abundance and diet quality of a common woodland salamander in its northern range

**DOI:** 10.1007/s10530-023-03168-3

**Published:** 2023-09-26

**Authors:** Trevor Scott, Robert L. Bradley, Patrice Bourgault

**Affiliations:** Département de Biologie, Université de Sherbrooke, Sherbrooke, QC Canada

**Keywords:** Gastric lavage, Non-native earthworms, *Plethodon cinereus*, Population density, Salamander diet, Visible implant elastomer

## Abstract

Non-native earthworms found in Eastern Canada substantially affect soil properties and plant diversity, but less is known about their impacts on higher faunal species. We investigated the effects of non-native earthworms on populations of *Plethodon cinereus*, a common woodland salamander. We hypothesized that earthworms could adversely affect *P. cinereus* by consuming the forest floor, thereby decreasing soil moisture and the abundance of native preys. Conversely, earthworms could positively affect *P. cinereus* by providing refuge in their abandoned burrows and by being a novel prey. We installed 25 coverboards in 38 mature sugar maple (*Acer saccharum*) forests, 24 of which were earthworm-free. Over the next two years, we monitored earthworm and salamander populations using hot mustard extractions and visible implant elastomers, respectively. At a subset of four sites, two with and two without earthworms, we determined salamander diets in the spring (May–June), summer (July–August) and fall (September–October) seasons, using gastric lavage techniques. Forest floor depth decreased, whereas population density, body size and total prey volume of *P. cinereus* increased, with earthworm abundance. Earthworms, which are soft-bodied and nutritious prey, composed most of the salamander diet at sites with earthworms, volumetrically accounting for > 50% of total prey volume. Despite this, we found fewer prey items in the stomach of salamanders at earthworm-invaded sites, indicating that salamanders are getting a higher caloric intake per feeding while expending less energy. We conclude that non-native earthworms have a net beneficial effect on *P. cinereus* populations in Eastern Canada, mainly by improving diet quality.

**Supplementary Information:**

The online version contains supplementary material available at 10.1007/s10530-023-03168-3.

## Introduction

Ancient earthworm populations that were once native to Eastern Canada were extirpated during the Wisconsin glacial period (ca. 75,000 to 11,000 years ago), during which time the Laurentide ice sheet covered the entire region (Hopfensberger and Hamilton 2015). Consequently, all earthworm species that are present today are non-native and were introduced by European settlers through the importation of soil and plant material over recent centuries (Gates 1961; Reynolds 1977). With a maximum migration rate of 5–10 m yr^−1^ (Marinissen and van den Bosch 1992), it is unlikely that earthworms could have dispersed through new habitats as rapidly as they have without human facilitation. This is supported by substantial evidence that the dispersal of earthworms into new habitats within Eastern Canada is closely linked to the transport of juveniles and cocoons in the treads of tires and shoes, as well as to anglers dumping live worm bait around fishing lakes (Drouin et al. 2016; Fugère et al. 2017). Consequently, earthworm populations are commonly abundant in urban and agricultural landscapes, and in some forest ecosystems with high human activities. In the southern regions of the province of Quebec (Canada), remote forested areas are generally earthworm-free whereas those used for recreation or commercial logging are generally colonized by earthworms. This situation allows us the opportunity to study the impacts of non-native earthworms on ecological processes, by comparing similar forests where earthworms are either present or absent. Most studies of this sort have focused on the impacts of non-native earthworms on soil properties (e.g., Dempsey et al. 2011; Resner et al. 2015; Drouin et al. 2016) or plant communities (e.g., Hale et al. 2006; Drouin et al. 2014; Craven et al. 2017). Fewer studies (e.g., Maerz et al. 2005; Ransom 2012) have investigated the effects of non-native earthworms on the demographics, diet, or body condition of higher faunal species such as soil surface-dwelling vertebrates. If such effects can be demonstrated, it would suggest that non-native earthworms play an important link between below and aboveground food webs.

One such vertebrate species that could potentially interact with non-native earthworms is the eastern red-backed salamander (*Plethodon cinereus* Green). This woodland salamander is commonly found in sugar maple (*Acer saccharum* Marsh.) forests of Eastern Canada, where it is an important animal group in terms of total biomass per land area (Burton and Likens 1975). It is a predator of forest floor-dwelling micro-invertebrates (Walton 2006, 2013), thereby regulating their numbers with attendant effects on various soil properties (Neupane et al. 2015). By the same token, the eastern red-backed salamander is prey to larger vertebrate species, which has led to various antipredator adaptations such as exhibiting color polymorphism (Venesky and Anthony 2007) and responding to chemical cues (Sullivan et al. 2002). While the eastern red-backed salamander is fully terrestrial, it requires a moist environment to enable its cutaneous respiration and maintain the viability of its eggs. Accordingly, it is commonly found beneath or within the forest floor, underneath rocks, inside rotting woody debris, and in naturally occurring holes in the mineral soil (Bishop 1944). Thus, the eastern red-backed salamander occupies a common habitat with earthworms, allowing for possible interactions between these two animal groups.

Non-native earthworms could affect the growth, fitness and population size of eastern red-backed salamanders in several ways. Firstly, earthworms consume and remove the forest floor (Dempsey et al. 2011) thereby increasing the risk of desiccation and reducing mobility. Removal of the forest floor may also reduce the abundance of prey for the eastern red-backed salamander and possibly increase its risk of being predated by larger vertebrates (Migge-Kleian et al. 2006; Maerz et al. 2009). Conversely, the presence of earthworms may bring about beneficial effects to eastern red-backed salamanders. For example, abandoned earthworm burrows can offer a refuge, thereby lowering the risk of predation and buffering the effects of harsh climatic conditions (Cáceres-Charneco and Ransom 2010; Ransom 2011). Also, earthworms may become a novel prey for eastern red-backed salamanders, while being more voluminous and nutritious than the typical micro-invertebrates that make up their native diets (Maerz et al. 2005). Thus, the studies that have explored non-native earthworm × salamander interactions have evoked mechanisms that have both positive and negative effects on the growth, fitness, and population size of eastern red-backed salamanders. However, these past studies present a few caveats. For example, none of the prior in situ studies (i.e., Maerz et al. 2005, 2009; Brunges et al. 2020) included control sites that were completely earthworm-free. Among these, only one study (Brunges et al. 2020) directly related earthworm to salamander abundances, while the others only provided indirect evidence for such a relationship (e.g., relating earthworm abundance to salamander prey abundance: Maerz et al. 2009). Thus, the net effect of non-native earthworms on eastern red-backed salamanders remains unclear.

One approach to better understand the relationship between non-native earthworms and the eastern red-backed salamander is to investigate the effects of individual earthworm feeding guilds. The most basic categorization of these feeding guilds is to sort earthworm species according to three ecotypes, namely the anecic, endogeic and epigeic earthworms (Hendrix 1995). Anecic earthworms create deep vertical burrows and feed on large volumes of leaf litter, which they pull into their burrows (Hendrix 1995). In Eastern Canada, anecic earthworms have the largest body size among the three feeding guilds (Edwards and Bohlen 1996) and contribute greatly to the reduction of the forest floor (Edwards and Bohlen 1996). Mature individuals are generally too large to be prey for eastern red-backed salamanders, however, their abandoned burrows could offer a useful refuge (Ransom 2012). For their part, endogeic earthworms create networks of horizontal burrows in the upper mineral soil horizons. Because they are generally smaller than anecic earthworms (Edwards and Bohlen 1996), they are less likely to create habitable burrows for eastern red-backed salamanders. Moreover, endogeic earthworms are not expected to degrade the eastern red-backed salamander’s habitat to the same extent as anecic species, because they are not active in removing fresh litter from the soil surface (Edwards and Bohlen 1996; Maerz et al. 2009). As for epigeic earthworms, these species live at the soil’s surface under rocks and coarse woody debris, they consume fresh leaf litter, and they are generally the smallest in size among the three feeding guilds (Edwards and Bohlen 1996). Epigeic earthworms thus contribute to the reduction of the forest floor, but they are probably the most visible and accessible as prey for eastern red-backed salamanders (Brunges et al. 2020). Thus, these three feeding guilds differ in terms of their effects on forest floor removal, creation of habitable burrows, and provision of prey to the eastern red-backed salamander.

In this article, we report on a study in which we monitored the relative abundance of anecic, endogeic and epigeic earthworm species across 38 mature sugar maple forest sites in southern Quebec, Canada. All sites were similar in tree species composition, tree age, drainage class, elevation, climate, and soil texture. The 38 sites were chosen to represent a gradient in earthworm abundance, with approximately two-thirds of sites being earthworm-free. Over two years, we monitored eastern red-backed salamander abundances as well as salamander body size and diet. Our objectives were to (1) evaluate the net effect of invasive earthworms on the demography of the eastern red-backed salamander in its northern range, and (2) evaluate the importance of exotic earthworms as a novel prey for the eastern red-backed salamander.

## Materials and methods

### Study sites

The study was conducted within a 100 km radius around the city of Sherbrooke (45° 24′ 16″ N and 71° 53′ 18″ W), located in the province of Quebec, Canada. The mean annual temperature of this area is 5.6 °C, with a mean monthly low of −10.6 °C in January and a mean monthly high of 19.6 °C in July (Government of Canada 2022). The mean annual precipitation is distributed as 940 mm of rainfall and 207 cm of snowfall (Government of Canada 2022). According to the forest ecological classification system of Quebec, the western part of our sampling area lies within the sugar maple–basswood bioclimatic domain whereas the eastern part lies within the sugar maple–yellow birch bioclimatic domain (Saucier et al. 1998).

**Fig. 1:**
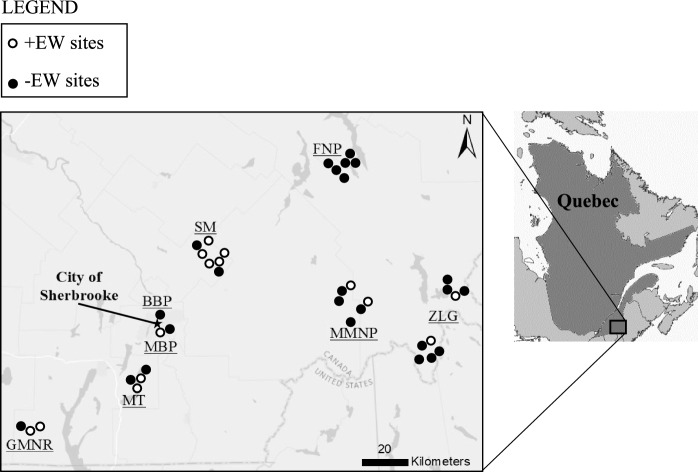
Geographic location of the study area within the province of Quebec (*right frame*) and each of the 38 study sites indicated by dots (*left frame*). Sites are grouped within eight different zones: Green-Mountain Nature Reserve (GMNR), Massawippi Trails (MT), Mont-Bellevue Park (MBP), Bois-Beckett Park (BBP), Stoke Mountains (SM), Frontenac National Park (FNP), Mont-Mégantic National Park (MMNP), and Controlled Harvesting Zone Louise-Gosford (ZLG)

In spring 2019, we located 38 forest stands within the study area (Fig. 1) using QGIS software Version 3.6.2 (QGIS Development Team 2022). Site selection was based on the following criteria: dominant tree species (> 80% sugar maple), stand size (> 5 ha), slope (< 30%), drainage (good to moderate), stand age (> 50 y-old), canopy cover (> 60%), and distance to the nearest road (< 1 km). Selected sites were located no less than 1 km apart to ensure that salamander populations were independent of one another. At a central location within each forest stand, we established a (30 × 30) m^2^ sampling plot to be used for earthworm and salamander sampling over the next two years.

### Soil properties

In summer 2019, we measured the depth of the organic forest floor at the four corners of each sampling plot and computed the mean depth for each plot. Surface mineral soil samples from each corner (0–30 cm depth) were then collected and combined into one bulk sample per plot. These were sieved through a 5 mm wire mesh, gently homogenized by hand mixing, and transferred into plastic bags. Bulk mineral soil samples were placed under ice packs in a cooler and transported to the laboratory, where they were stored at 4 °C until analyzed.

Mineral soil samples from each plot were characterized according to water holding capacity, pH, texture, % organic matter, total C and N, mineralizable N and microbial biomass. Water holding capacity was calculated as the gravimetric moisture content of soil subsamples (ca. 20 g) that had been saturated with water and left to drain for 24 h on cellulose filter papers suspended in plastic funnels. Soil pH, in deionized water as well as in 1 M KCl solution, were measured in soil slurries (soil:liquid = 1:2.5) using a hydrogen electrode. Soil texture was determined using the hydrometer method (Bouyoucos 1962). Percent soil organic matter was measured by mass loss on ignition, after burning dried subsamples (ca. 50 g) in a muffle furnace at 400 °C for 6 h. Total C and N were measured by grinding oven-dried subsamples with a ball mill, encapsulating 5–10 mg of the ground soil in Sn sheets, and analyzing these by high-temperature combustion and thermo-conductometric detection using a Carlo Erba NC2500 elemental analyzer (Carlo Erba Ltd., Val-de-Reuil, France). Mineralizable N was determined by incubating field moist subsamples (ca. 15 g) at 22 °C for 30 d and extracting these with 100 mL of 1 M aqueous KCl solution; the filtered extract solutions were colorimetrically analyzed for NH_4_^+^-N (salicylate–nitroprusside-hypochlorite assay) and NO_3_^−^-N (Cd reduction-sulphanilamide assay) using an Astoria2 Autoanalyzer (Astoria-Pacific, Clackamas, OR). Microbial biomass was measured by substrate induced respiration (Anderson and Domsch 1978) according to the protocol described by Lapointe et al. (2005), using a Varian 431-GC gas chromatograph (Varian Analytical Instruments, Walnut Creek, CA).

### Earthworm species and abundances

Earthworm communities at each site were sampled on four occasions, in June and July 2020 and in May and September 2021. At each sampling date, two technicians collected epigeic earthworm species found under woody debris, rocks and other coarse fragments, over a period of 15 min. They then sampled endogeic and anecic species at eight random locations around the perimeter of each plot, using the hot mustard extraction method (Lawrence and Bowers 2002). This was accomplished by removing the forest floor and excavating the mineral soil over a (30 × 30) cm^2^ area, down to 30 cm depth. The excavated soil was placed on a plastic sheet and hand sorted to collect all earthworms. The technicians then poured 2 L of hot mustard solution (10 g L^−1^) into each hole and waited 15 min. to expel deeper-dwelling earthworms (Chan and Munro 2001). All earthworms collected in each plot were fixed in a solution of 10% formaldehyde and transported to the laboratory for identification based on Reynold’s key (Reynolds et al. 2017). Juvenile earthworms (i.e., those lacking a clitellum) could not be identified, therefore we assumed that the species relative abundance of these juveniles to be the same as adults for a given site. Given that different earthworm species vary in size, we converted the abundance of each species to an estimate of ash-free dry mass (AFDM) using allometric equations gleaned from the literature, as per Cameron et al. (2021) (Supplementary Table S1).

### Salamander abundance, body size and diet

In the summer of 2017, 950 coverboards measuring (50 × 25 × 5) cm^3^ were cut from untreated pine wood (Hesed 2012), then placed in a forested area to weather for two years. In June 2019, 25 coverboards were installed at each site in a 5 × 5 grid, with a 5 m distance separating each coverboard. Over the next two years (2020 and 2021), the relative abundance of eastern red-backed salamanders was measured nine times during their active season (May to October). Sampling consisted of lifting each coverboard and collecting all eastern red-backed salamanders in a plastic bag containing a small amount of deionized water. We measured each individual’s snout-vent length (SVL) and tagged newly captured individuals with a unique visible implant elastomer (Northwest Marine Technologies, Shaw Island, USA) for future identification as well as to avoid including multiple counts of the same individual (Nauwelaerts et al. 2000; Ralston Marold 2001). Elastomer tags consisted of one to four different colors that we injected subcutaneously at ventral locations near the base of one to four limbs per individual, using a hypodermic needle (Heemeyer et al. 2007). The color codes were generated using the VIE Color Code Generator program (Northwest Marine Technologies, Shaw Island, USA). The tagged salamanders were then released directly beside the coverboard from which they had been captured. Given the two-year sampling period and the coverboard sampling design, our data did not comply with the basic assumptions of capture-mark-recapture models to estimate population size (i.e., no immigration or emigration, no births or deaths, etc.). We thus calculated relative salamander abundance as the number of unique individuals (i.e., unique IDs) captured over the nine sampling dates.

To test the effect of earthworms on eastern red-backed salamander diets, we intensively sampled two sites where earthworms were abundant and two sites that were earthworm-free. In 2021, we visited each of these four sites in spring (May–June), summer (July–August) and fall (September–October), coinciding with the active season of the eastern red-backed salamander. Given that this species is a nocturnal feeder that is most active when soils are moist, we sampled each site between 5:00 and 10:00 a.m. following or during a rainfall event. We sampled specimen found under rocks and coarse woody debris within an area of 10 m beyond the perimeter of the plot so as not to disturb the population of salamanders under the coverboards. The captured salamanders (~ 90 per site) were temporarily held in a sealed plastic bag containing a small amount of deionized water. Each individual was immobilized in a non-bleached pre-moistened paper towel, its mouth was held open using a wooden toothpick, and a medical-grade soft plastic catheter (I.D. 0.38 mm × O.D. 1.02 mm) was inserted into its esophagus (Bondi et al. 2015). The catheter was attached to a 6 mL syringe filled with deionized water, which was gently pushed into the salamander’s stomach in two 3 mL increments. The regurgitated stomach content was fixed in a vial containing a 90% ethanol solution and returned to the laboratory for identification. Due to the size of the catheter, diet samples were only collected from individuals with a SVL > 34 mm. After performing the gastric lavage procedure, salamanders were returned directly beside the cover object from which they were collected. After returning to the laboratory, each gastric sample was magnified (7.3× to 120×) under a model M165FC digital microscope (Leica Microsystems, Wetzlar, Germany). Individual prey items were counted and identified to the lowest possible taxonomic level using keys provided by Marshall (2017). The volume of each prey item was estimated using OMERO (Version 5.11.0) microscope-image processing software (Glencoe Software, Inc., Washington, USA).

### Statistical analyses

We performed all statistical analyses using R Core Team (2019) and RStudio Version 1.2.x RStudio Team (2019) software. The abundance of earthworms was computed as either a categorical (i.e., presence vs. absence) or numerical (i.e., collected earthworm biomass) predictor variable, according to the statistical test that we used.

We compared the value of each soil property in earthworm-invaded vs. earthworm-free sites using Student’s *t*-tests. We used generalized linear models (GLM) coded in the *MASS* R package (Venables and Ripley 2002) to test the effect of total earthworm biomass on forest floor depth as well as on eastern red-backed salamander counts. We then used three-factor GLM models to test the effects of the three earthworm feeding guilds (i.e., epigeic, endogeic and anecic) on the same two response variables. Each of these GLM models used a negative binomial distribution of error terms to account for overdispersion in the data.

We used similar GLM models to test the effects of earthworm biomass on eastern red-backed salamander body size (SVL). Here, we used a Poisson distribution of error terms because we only included adult specimen with SVL ≥ 34 mm (Sayler 1966), which yielded a truncated right-skewed data set. For specimen that were captured more than once, we used the first recorded SVL measurement as the dependent variable. Again, we first used a one-factor model to test the effect of total earthworm biomass, followed by a three-factor model to test the effect of the three earthworm feeding guilds. We also compared SVL between earthworm-invaded vs. earthworm-free sites using the non-parametric two-sample Mann–Whitney *U* test, given that the SVL data were not normally distributed.

For each of the three diet sampling periods, we compared the regurgitated stomach content of salamanders in earthworm-invaded vs. earthworm-free sites using multivariate analysis of variance (MANOVA) tests. The response variables for these tests were the mean volume of prey groups that accounted for more than 5% of total prey volume. Prey groups that accounted for less than 5% of total prey volume were combined into one single group, which we labelled as “other”. For each of the three sampling periods (i.e., spring, summer and fall), we performed Student’s *t*-tests to compare the mean number of prey items, the mean total prey volume and the mean non-earthworm prey volume of eastern red-backed salamanders captured on earthworm invaded vs. earthworm-free sites.

## Results

Our earthworm survey revealed 19 sites with the presence of earthworms (EW+) and 19 sites that were earthworm-free (EW-). Fourteen of the 19 EW+ sites had total AFDM values in the 5–78 g range whereas five sites were in the 1–5 g site^−1^ range. These five low-abundance sites showed similar properties (i.e., forest floor depth and salamander counts) as EW- sites and were therefore classified as EW- sites. All of the remaining 14 EW+ sites included epigeic and endogeic earthworm species whereas anecic species were present on only seven sites.

**Fig. 2:**
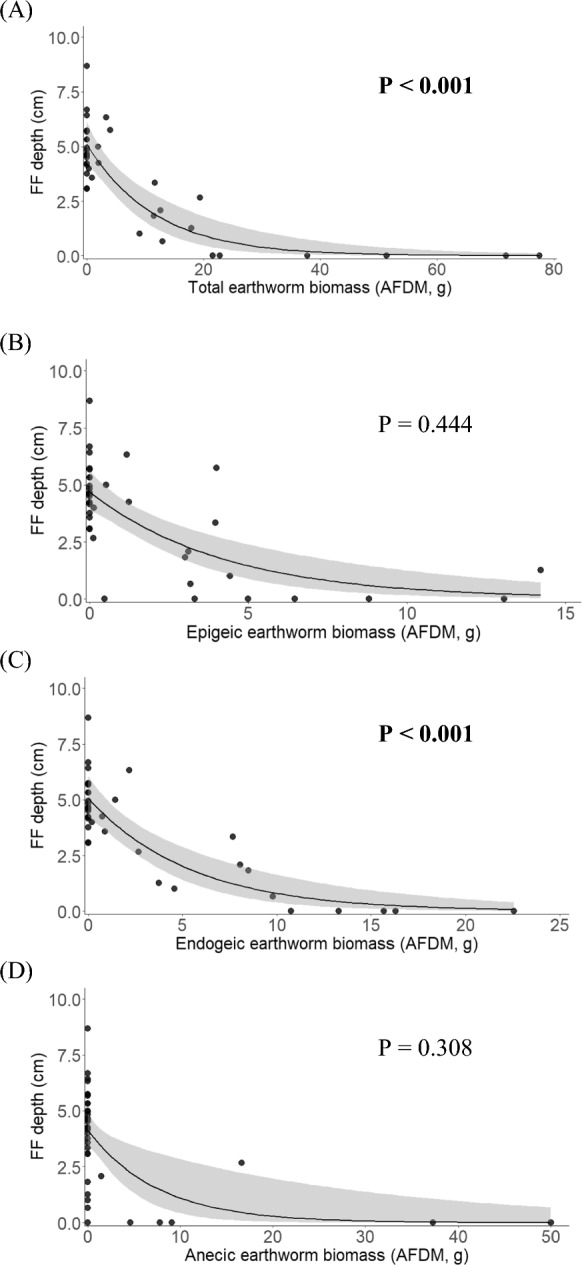
The effect of **A** total, **B** epigeic, **C** endogeic, and **D** anecic earthworm abundance, expressed as ash-free dry mass (AFDM), on forest floor (FF) depth. Shaded areas represent 95% confidence intervals. *P* values < 0.05 are shown in bold print (N = 38)

**Fig. 3:**
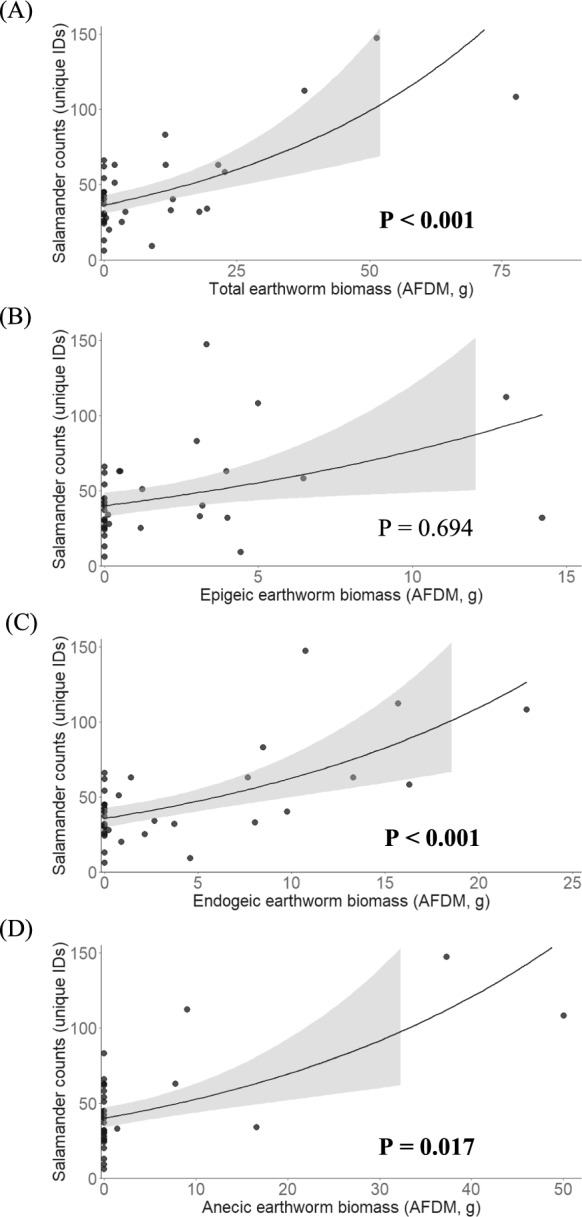
The effect of **A** total, **B** epigeic, **C** endogeic, **D** anecic earthworm abundance, expressed as ash-free dry mass (AFDM), on Eastern red-backed salamander counts. Shaded areas represent 95% confidence intervals. *P* values < 0.05 are shown in bold print (N = 38)

**Fig. 4:**
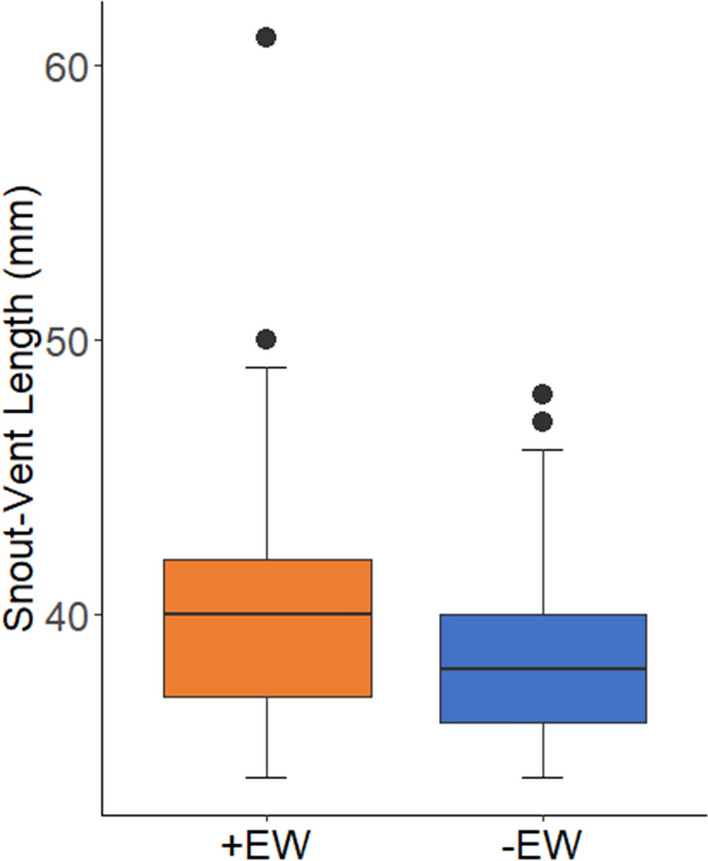
Box and whisker plot comparing of mean snout-vent length (SVL) of adult (SVL ≥ 34 mm) Eastern red-backed salamanders on earthworm-invaded (EW+ , n = 668) and earthworm-free (EW−, n = 522) sites. Means were compared using a Mann–Whitney *U*-test (*P* < 0.001)

Among the various mineral soil properties that we measured, EW+ sites had higher pH in water (*P* = 0.004), pH in KCl (*P* < 0.001), total-N (*P* = 0.014), mineralizable-N (*P* = 0.001) and microbial biomass (*P* < 0.001) than EW− sites (Table 1; raw data shown in Supplementary Table S2). We found negative effects of total earthworm biomass (*P* < 0.001) and endogeic earthworm biomass (*P* < 0.001) on forest floor depth (Fig. 2A and C). Conversely, we found positive effects of total (*P* < 0.001), endogeic (*P* < 0.001) and anecic (*P* < 0.017) earthworm biomass on eastern red-backed salamander counts (Fig. 3A, C and D). We did not find any meaningful relationship between earthworm biomass (i.e., total biomass and individual feeding guilds) and SVL, although the slope within each model was positive (data not shown). A Mann–Whitney *U* test revealed a larger (*P* < 0.001) mean SVL on EW+ than on EW− sites (Fig. 4; raw data shown in Supplementary Fig. S1).

**Table 1:** Physicochemical properties of mineral soil (0–30 cm) at earthworm-invaded (+EW, n = 14) and earthworm-free (−EW, n = 24) sites

Soil property	+EW	−EW	t-Value	*P* > 1 ***t*** |
Water holding capacity (%)	94.6 ± 0.3	85.2 ± 0.2	1.349	0.186
pH (water)	4.9 ± 0.3	4.6 ± 0.2	3.113	**0.004**
pH (1 M KCl)	4.3 ± 0.3	3.9 ± 0.2	4.249	** < 0.001**
Sand (%)	61.9 ± 14.1	58.8 ± 11.3	0.753	0.753
Clay (%)	4.0 ± 2.0	3.1 ± 1.7	1.330	0.192
Organic matter (%)	10.7 ± 3.2	9.8 ± 2.7	0.850	0.401
Total C (%)	5.3 ± 1.6	4.9 ± 1.4	0.388	0.400
Total N (%)	0.4 ± 0.1	0.3 ± 0.1	2.581	**0.014**
Mineralizable N (ug g^−1^)	28.6 ± 9.0	19.0 ± 6.6	3.769	**0.001**
Microbial biomass (ug Cmic g^−1^)	355.2 ± 124.6	223.1 ± 85.7	3.870	** < 0.001**

Values represent the mean ± 1 S.D. for each mineral soil property. Means were compared using Student's *t*-tests. Significant *P* values (< 0.05) are shown in bold print

**Fig. 5:**
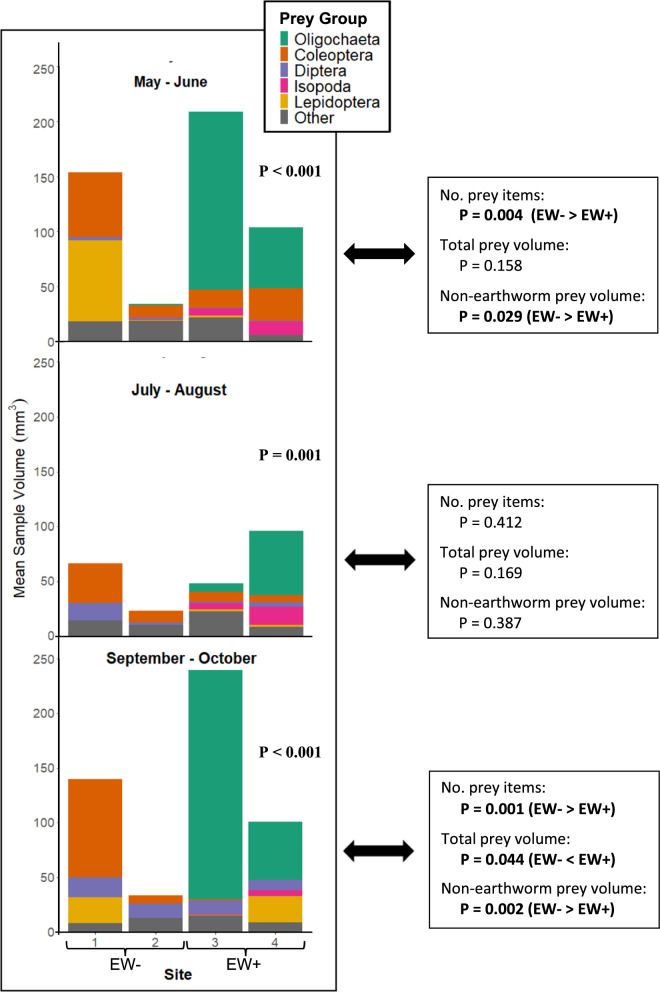
Mean prey volume of Eastern red-backed salamanders collected in two earthworm-free (EW−) and two earthworm-invaded (EW+) sites during spring (May–June), summer (July–August) and fall (September–October) sampling periods. At each sampling period, a total of 30–35 individuals were sampled at each site. The reported P value in each frame is the result of a MANOVA test comparing the diets of salamanders on EW− (1 and 2) and EW+ (3 and 4) sites. These tests were based on prey groups that accounted for more than 5% of total prey volume (Supplementary Table S3). The prey group “Other” includes Gastropoda, Hymenoptera, Myriapoda, Arachnida (Araneae and Opiliones), Arachnida (Acariform and Parasitiform) and Collembola. Boxes on the right report the results of Student *t*-tests comparing the mean number of prey items, the mean total prey volume and the mean non-earthworm prey volume for each sampling period

In each of the three sampling periods, MANOVA tests revealed distinct salamander diets on earthworm-invaded compared to earthworm-free sites (*P* < 0.001). Most notably, earthworms were an important prey on EW+ sites, comprising 50–85% of total prey volume on five out of six site × date combinations (Fig. 5; raw data shown in Supplementary Table S3). Results from Student’s *t*-tests revealed fewer prey items per salamander on EW+ than on EW− sites during the spring (*P* = 0.004) and fall (*P* = 0.001) sampling periods (Fig. 5). However, total prey volume was higher on EW+ sites, especially in the fall period (*P* = 0.044). The non-earthworm prey volume was higher on EW− sites during the spring (*P* = 0.029) and fall (*P* = 0.002) sampling periods.

## Discussion

The higher microbial biomass, higher total and mineralizable-N, as well as higher pH that we observed in the mineral soil of EW+ plots is consistent with the comminuting and burrowing activities of earthworms that accelerate nutrient cycling and generally improves soil fertility (e.g., van Groenigen et al. 2014). However, the soil property that concerned us the most in terms of potential impacts on eastern red-backed salamander populations was the depth of the organic forest floor. Forest floor depth in EW- plots was roughly 3–7 cm deep, but declined sharply as total earthworm abundance increased, in accordance with results from prior studies (Frelich et al. 2006; Suárez et al. 2006; Hale et al. 2008). The fact that endogeic earthworms was the only feeding guild to show a strong negative relationship with forest floor depth came as a surprise. The literature tells us that only epigeic and anecic species are active in the comminution of leaf litter (Addison 2009), whereas endogeic species are not expected to participate in the removal of the forest floor (Shipitalo and Edwards 1998; Maerz et al. 2009). However, the broad classification scheme of earthworms into three feeding guilds may be too simplistic, as some species display traits that can be assigned to two of these categories at once (Bottinelli et al. 2022). For example, the earthworm *L. rubellus* (Hoffmeister) found across our EW+ plots is conventionally classified as an endogeic species, although it purportedly feeds on forest floor organic matter and is thus better defined as an “epi-endogeic” species (Addison 2009). Accordingly, Suárez et al. (2006) found that soil inhabited by two endogeic species (*L. rubellus* and *Octolasion tyrtaeum* Savigny) had substantially less forest floor than a neighbouring soil inhabited by an anecic species (*L. terrestris* Linnaeus). Some authors have also reported that endogeic earthworms participate indirectly to litter decomposition, by stimulating other soil faunal groups (Bonkowski et al. 2001). Hence, it is reasonable to presume that all earthworm feeding guilds, including endogeic species, could contribute to a reduction of the forest floor and, with that, a modification of habitat for eastern red-backed salamander populations.

A reduction of the forest floor due to earthworms may have several negative effects on the eastern red-backed salamander. Firstly, the water storage capacity of organic forest floors in temperate deciduous forests is several times greater than that of mineral soil horizons (Leuschner 1998). As a lungless amphibian, the eastern red-backed salamander relies on the humidity of the forest floor to buffer dry weather conditions and maintain a moist microclimate that enables its cutaneous respiration during its movements (Feder 1983). This is supported by field data showing a strong relationship between forest foor moisture and the abundance of this salamander species (Heatwole 1962). Besides reducing the forest foor, earthworms also create macropores in the mineral soil that provide preferential flow pathways that allow surface water to infiltrate deeper into the soil (van Schaik et al. 2014). Thus, even on EW+ sites where the forest floor is still present, the burrowing activities of earthworms will likely reduce moisture because of increased drainage. Secondly, a reduction of the forest floor coincides with a reduction of soil and litter-dwelling microarthropods, such as oribatid mites and collembola, which constitute the typical native prey of eastern red-backed salamanders (Migge 2001; Stuczka et al. 2016). Maerz et al. (2009) proposed that this reduction in prey may cause a decline in eastern red-backed salamander populations. Thirdly, the forest floor provides cover for eastern red-backed salamanders during displacements and when foraging, making them less visible to predators (Walls 1995). In spite of these plausible negative effects of forest floor removal, our data showed an increase in eastern red-backed salamander populations with increasing earthworm abundance. This suggests that exotic earthworms are providing some other benefits to salamander populations that more than compensate for any detrimental effect related to forest floor loss.

A first mechanism that may be driving the increase in eastern red-backed salamander populations is that of an improved diet in the presence of earthworms (Maerz et al. 2005; Ransom 2012). Our data provide strong support for this argument, given that we found higher prey volume in the stomachs of salamanders on EW+ plots compared to those on EW− plots. Furthermore, earthworms generally comprised 50–80% of total prey volume on EW+ plots, with only one exception (i.e., site #3 in July–August). Earthworms are a higher quality prey than the typical native diet of eastern red-backed salamanders (i.e., small arthopods) because they are nutrient-rich, soft-bodied and easier to digest (Maerz et al. 2005; Anthony et al. 2017). Moreover, earthworms accounted for a smaller percentage of total prey items relative to their percentage of total prey volume. This indicates that eastern red-backed salamanders on EW+ plots are getting a higher caloric intake per feeding while expending less energy. This, in turn, could explain the greater body size (SVL) that we observed on EW+ plots. Wise and Jaeger (2021) demonstrated that maternal body size of eastern red-backed salamanders was a good predictor of reproductive success. More specifically, they found that maternal SVL was positively related to clutch size, to offspring SVL as well as to offspring survival. This overall increase in fitness could explain the higher abundances of eastern red-backed salamanders that we observed on EW+ plots.

A second mechanism that may favor an increase in eastern red-backed salamanders on EW+ plots is their possible use of anecic earthworm burrows as a refuge. Adult anecic earthworms, such as *L. terrestris*, are generally too large to serve as prey for the eastern red-backed salamander (Ransom 2012). However, they do create extensive networks of vertical burrows that are size-appropriate for use by eastern red-backed salamanders (Cáceres-Charneco and Ransom 2010). Plethodontid salamanders in general rely on the availability of suitable pre-existing burrows, as they are generally unable to dig their own (Heatwole 1960). Rather, they occupy naturally occurring fissures in the soil, root hollows left after the decay of dead roots, and burrows created by other organisms (Heatwole 1960; Ransom 2011). These burrows can be used to avoid their predators such as birds (Kraemer and Adams 2014), small mammals (Brodie et al. 1979) and reptiles (Ransom 2011). In addition to predator evasion, these burrows can also protect eastern red-backed salamanders from adverse weather conditions. During warmer months, the soil surface becomes dry, especially in forest environments with little forest floor. Eastern red-backed salamanders can therefore use anecic earthworm burrows to retreat underground where it is cool and moist (Feder 1983). Conversely, during colder winter months, they can use these burrows to avoid freezing surface temperatures and to enter a state of torpor (Ransom 2012). At its deepest, the soil frostline in Southern Quebec extends to around 1 m below the surface (Transport Québec 2021), whereas anecic earthworm burrows may be up to 3 m deep (Lee 1985). Although our study did not test for the use of earthworm burrow by eastern red-backed salamanders, there is corroborative evidence in the fact that the three sites with the highest salamander abundances were among the seven EW+ sites where anecic earthworms were present.

One potential bias that we considered in our study was whether a reduction or elimination of the forest floor by earthworms may cause eastern red-backed salamanders to gravitate towards the cover boards, or to spend more time there. While we have no way of knowing this, it is a fact that the forest floor had already been reduced or eliminated for some time on earthworm-invaded sites. If the presence of a forest floor was important for maintaining eastern red-backed salamander populations, then we would expect extremely small salamander counts on our earthworm invaded sites. It is unlikely that adding the cover boards would have prompted a long-range migration of salamanders towards these boards, given their very small home range. This is supported by data from Kleeberger and Werner (1982) who showed that most eastern red-backed salamanders are found in mineral soil burrows or within rotting logs, whereas their presence in the forest floor is only transient. These authors also reported that the average daily movement of *P. cinereus* was 0.4 m, which could increase to about 1.0 m during periods of strong precipitation, with a nearly 100% return rate to their permanent refuge. Hence, we believe that eastern red-backed salamander counts under coverboards is a reliable estimate of relative abundances across sites.

Our study adds to the growing body of evidence that the consequences of earthworm invasions are extensive, not only having impacts on soil microbial and plant communities but extending beyond to at least the level of soil-dwelling vertebrate fauna. We found that exotic earthworm populations, when present, constitute the most important prey for eastern red-backed salamanders in southern Quebec, coinciding with higher salamander body size and population densities. This opens the discussion on whether the introduction of exotic earthworms alters further trophic interactions by shifting ecosystem energy flow towards yet higher consumers. Burton and Likens (1975) estimated that the biomass of eastern red-backed salamander populations in hardwood forests of the neighbouring state of New Hampshire (USA) was approximately twice that of birds and approximately equal to that of small mammals. Thus, the eastern red-backed salamander is potentially an important prey to passeriform birds, small mammals (e.g., raccoons, skunks and moles), amphibians and reptiles (e.g., frogs and snakes). Further research should therefore strive to determine whether exotic earthworms create cascades of ecological change such as diversifying adaptive traits of predators (Broderson et al. 2015), shifting spatial and temporal patterns of prey abundance (Maerz et al. 2005) and regulating nutrient cycling patterns (Milanovich and Peterman 2016).

### Supplementary Information

Below is the link to the electronic supplementary material.

Supplementary file1 (PDF 83 KB)

Supplementary file2 (PDF 85 KB)

Supplementary file3 (PDF 69 KB)

Supplementary file4 (PDF 176 KB)

## Data Availability

The data underlying this article will be shared upon reasonable request to the first or corresponding author.
