# Supplementary material for: Non-native earthworms increase the abundance and diet quality of a common woodland salamander in its northern range
Source: Biol Invasions. 2023 Sep 26;26(1):187–200. doi: 10.1007/s10530-023-03168-3 (PMC10781809; doi:10.1007/s10530-023-03168-3)
Supplement: Supplementary file 1 — Supplementary file1 (PDF 83 KB) [file 10530_2023_3168_MOESM1_ESM.pdf]

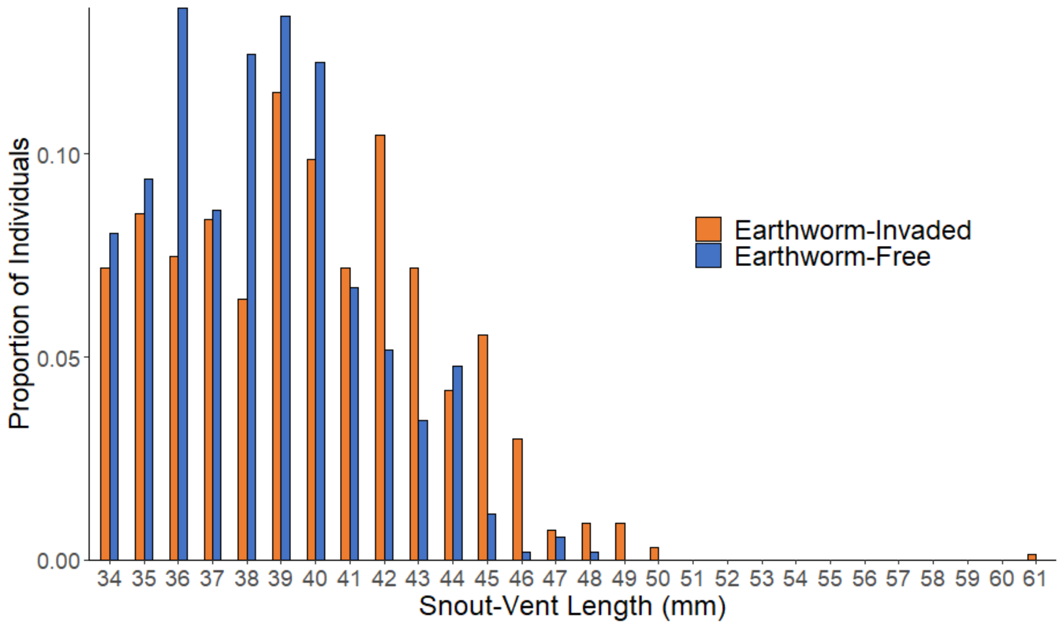

Figure S1. Body size distribution of Eastern red-backed salamanders at earthworm-invaded (n = 668) and earthworm-free (n = 522) sites. This figure considers only adult salamanders with a snout-vent length (SVL) ≥ 34 mm. Each bar represents the proportion of all salamanders within a size class.
