# Supplementary material for: Non-native earthworms increase the abundance and diet quality of a common woodland salamander in its northern range
Source: Biol Invasions. 2023 Sep 26;26(1):187–200. doi: 10.1007/s10530-023-03168-3 (PMC10781809; doi:10.1007/s10530-023-03168-3)
Supplement: Supplementary file 2 — Supplementary file2 (PDF 85 KB) [file 10530_2023_3168_MOESM2_ESM.pdf]

**Table S1.** Allometric equations used to convert the abundance of sampled earthworm species into estimates of ash-free dry mass (AFDM).

| Species Name | Length (mm) | AFDM conversion equation | AFDM (g) |
|---|---|---|---|
| *Allobophora chlorotica* | 30–70 | ln(AFDM) = 2.285*ln (Length)-11.905 | 0.0516 |
| *Aporrectodea calignosa* | 60–85 | ln(AFDM) = 2.285*ln (Length)-11.905 | 0.121 |
| *Aporrectodea longa* | 90–150 | ln(AFDM) = 2.285*ln (Length)-11.905 | 0.381 |
| *Aporrectodea rosea* | 25–85 | ln(AFDM) = 2.285*ln (Length)-11.905 | 0.064 |
| *Dendrobaena octaedra* | 17–60 | ln(AFDM) = 2.285*ln (Length)-11.905 | 0.028 |
| *Eiseniella tetraedra* | 30–60 | ln(AFDM) = 2.285*ln (Length)-11.905 | 0.041 |
| *Lumbricus castaneus* | 30–50 | ln(AFDM) = 2.285*ln (Length)-11.905 | 0.031 |
| *Lumbricus rubellus* | 50–150 | ln(AFDM) = 2.285*ln (Length)-11.905 | 0.251 |
| *Lumbricus terrestris* | 90–300 | ln(AFDM) = 2.285*ln (Length)-11.905 | 1.157 |
| *Octolasion tyrtaeum* | 25–130 | ln(AFDM) = 2.612*ln (Length)-13.019 | 0.191 |

[a] Earthworm species length provided by Reynolds and Reynolds (1992), and AFDM estimates by Hale et al. (2004).

[b] The most general conversion equation was used for A. chlorotica, E. foetida, E. tetraedra and F. playura montana, as Hale et al. (2004) did not provide conversion equations for these species.

[c] The species A. trapezoides, A. tuberculata and A. turgida were treated as A. calignosa, as per Pižl (2002) and Csuzdi and Zicsi (2003).
