# Supplementary material for: Non-native earthworms increase the abundance and diet quality of a common woodland salamander in its northern range
Source: Biol Invasions. 2023 Sep 26;26(1):187–200. doi: 10.1007/s10530-023-03168-3 (PMC10781809; doi:10.1007/s10530-023-03168-3)
Supplement: Supplementary file 3 — Supplementary file3 (PDF 69 KB) [file 10530_2023_3168_MOESM3_ESM.pdf]

Table S2. Geographic coordinates, and mineral soil physicochemical properties**.** See (Figure 1.) for the full form of abbreviated site zones.

Site Name	Latitude (DMS)	Longitude (DMS)	pH (H2O)	pH (KCl)	Moisture Content (%)	Total C (%)	Soil Texture	Mineralizable N (μg g^{-1})	Microbial biomass (mg C_{mic} g^{-1})
MT-1	45° 13' 26.4" N	72° 1' 4.8" W	4.89	4.04	37.76	3.22	Sandy Loam	7.03	222.08
MT-2	45° 14' 2.4" N	72° 0' 39.6" W	5.12	4.58	31.64	3.27	Sandy Loam	27.79	413.74
MT-3	45° 13' 44.4" N	72° 0' 43.2" W	5.35	4.81	43.41	4.10	Silt Loam	28.64	368.19
MT-4	45° 13' 58.8" N	72° 1' 22.8" W	4.46	3.92	37.13	3.58	Silt Loam	21.22	189.37
MBP-1	45° 22' 51.6" N	71° 54' 28.8" W	4.51	3.77	46.33	4.31	Silt Loam	14.48	236.14
MBP-2	45° 22' 37.2" N	71° 55' 1.2" W	5.27	4.87	56.48	5.16	Sandy Loam	24.32	268.64
BBP-1	45° 25' 4.8" N	71° 55' 30" W	4.03	3.62	53.34	7.01	Sandy Loam	26.48	273.69
SM-1	45° 33' 32.4" N	71° 44' 20.4" W	5.19	4.58	87.65	9.33	Loamy Sand	29.39	382.21
SM-2	45° 33' 3.6" N	71° 43' 44.4" W	4.83	4.00	36.21	3.07	Loamy Sand	6.20	169.49
SM-3	45° 33' 50.4" N	71° 43' 22.8" W	4.53	3.89	64.87	6.43	Loamy Sand	24.62	324.46
SM-4	45° 35' 24" N	71° 40' 12" W	5.15	4.45	50.94	4.98	Sandy Loam	20.59	329.01
SM-5	45° 34' 22.8" N	71° 42' 39.6" W	4.42	4.02	67.72	6.15	Sandy Loam	41.48	330.89
SM-6	45° 36' 46.8" N	71° 46' 33.6" W	4.08	3.61	45.61	8.52	Sandy Loam	20.66	199.14
SM-7	45° 37' 22.8" N	71° 43' 12" W	4.44	3.94	60.38	5.83	Loamy Sand	27.34	279.46
MMNP-1	45° 29' 13.2" N	71° 11' 52.8" W	4.26	3.87	62.34	5.63	Sandy Loam	18.72	161.00
MMNP-2	45° 29' 34.8" N	71° 10' 15.6" W	5.11	4.49	68.73	5.46	Silt Loam	23.39	290.89
MMNP-3	45° 27' 46.8" N	71° 5' 56.4" W	4.63	4.20	65.37	6.84	Sandy Loam	44.59	682.38
MMNP-4	45° 26' 13.2" N	71° 7' 48" W	4.73	4.23	48.36	4.27	Sandy Loam	21.96	149.38
MMNP-5	45° 24' 10.8" N	71° 10' 26.4" W	4.48	3.74	61.05	5.48	Sandy Loam	15.97	215.44
MMNP-6	45° 27' 36" N	71° 12' 54" W	4.64	3.88	40.15	3.44	Sandy Loam	17.37	162.46
ZLG-1	45° 29' 52.8" N	70° 46' 26.4" W	4.52	3.93	41.43	4.81	Sandy Loam	13.84	217.42
ZLG-2	45° 29' 13.2" N	70° 46' 19.2" W	4.43	4.00	44.01	4.25	Sandy Loam	16.55	180.74
ZLG-3	45° 28' 12" N	70° 43' 48" W	4.63	3.94	49.48	5.44	Sandy Loam	19.36	245.30
ZLG-4	45° 27' 54" N	70° 45' 28.8" W	4.64	4.04	41.22	3.01	Sandy Loam	16.68	129.75
ZLG-5	45° 17' 45.6" N	70° 53' 49.2" W	4.47	3.72	47.23	4.68	Silt Loam	36.43	433.53
ZLG-6	45° 20' 9.6" N	70° 52' 15.6" W	4.65	3.77	40.15	4.49	Silt Loam	29.92	384.91
ZLG-7	45° 19' 48" N	70° 50' 9.6" W	4.91	4.18	56.65	5.36	Sandy Loam	24.76	376.73
ZLG-8	45° 19' 30" N	70° 50' 49.2" W	4.29	3.65	29.54	2.88	Sandy Loam	22.33	223.83
ZLG-9	45° 20' 34.8" N	70° 51' 10.8" W	4.43	3.84	42.59	4.08	Loamy Sand	27.96	288.73
FNP-1	45° 49' 48" N	71° 14' 45.6" W	4.75	3.81	45.58	5.06	Silt Loam	12.27	15.12
FNP-2	45° 49' 37.2" N	71° 14' 20.4" W	4.48	3.74	54.99	6.98	Sandy Loam	17.24	286.88
FNP-3	45° 49' 15.6" N	71° 13' 55.2" W	4.36	3.86	50.82	5.43	Loam	18.18	168.56
FNP-4	45° 50' 45.6" N	71° 10' 40.8" W	4.79	4.19	63.35	6.34	Sandy Loam	24.11	198.94
FNP-5	45° 51' 7.2" N	71° 10' 58.8" W	4.84	4.26	49.59	4.64	Loamy Sand	15.07	202.26
FNP-6	45° 50' 16.8" N	71° 11' 13.2" W	4.72	4.10	66.92	5.55	Sandy Loam	20.65	198.48
GMNR-1	45° 6' 18" N	72° 26' 45.6" W	4.87	4.35	56.19	5.69	Sandy Loam	44.49	459.98
GMNR-2	45° 6' 28.8" N	72° 27' 32.4" W	4.8	4.30	37.06	3.61	Sandy Loam	16.23	242.56
GMNR-3	45° 6' 32.4" N	72° 25' 55.2" W	4.89	4.11	33.46	4.42	Sandy Loam	19.15	423.88
