# Supplementary material for: Non-native earthworms increase the abundance and diet quality of a common woodland salamander in its northern range
Source: Biol Invasions. 2023 Sep 26;26(1):187–200. doi: 10.1007/s10530-023-03168-3 (PMC10781809; doi:10.1007/s10530-023-03168-3)
Supplement: Supplementary file 4 — Supplementary file4 (PDF 176 KB) [file 10530_2023_3168_MOESM4_ESM.pdf]

Table S3. Raw data of Eastern red-backed salamander dietary samples collected over 3 sampling periods. Relative abundance is the occurrence of prey items from a given taxon relative to the total number of prey items collected. Relative volume is the proportion of the volume from a given taxon relative to the total volume of prey items.

May – June								
	- Earthworm				+ Earthworm			
Site	1		2		3		4	
Taxon	Rel. Abun. (%)	Rel. Vol. (%)	Rel. Abun. (%)	Rel. Vol. (%)	Rel. Abun. (%)	Rel. Vol. (%)	Rel. Abun. (%)	Rel. Vol. (%)
Araneae	4.3	1.0	1.2	5.7	2.3	0.6	0.9	0.9
Archaeognatha	---	---	---	---	---	---	---	---
Cent/Mill	2.7	1.3	4.1	22.9	2.7	0.7	0.4	0.4
Coleoptera	21.6	38.3	2.6	32.6	6.8	8.2	28.3	28.3
Collembola	11.8	0.5	61.8	12.6	24.4	0.7	1.0	1.0
Diplura	---	---	---	---	---	---	---	---
Diptera	5.1	1.8	2.0	5.6	2.3	0.2	1.0	1.0
Gastropoda (Slugs)	0.4	< 0.1	---	---	0.9	4.2	---	---
Gastropoda (Snails)	---	---	2.1	4.1	1.4	0.3	0.2	0.2
Hemiptera	---	---	0.3	3.2	---	---	0.7	0.7
Hymenoptera	10.6	6.0	1.0	2.2	0.9	< 0.1	0.3	0.3
Isopoda	---	---	0.8	0.8	10.0	3.2	11.9	11.9
Lepidoptera	2.4	48.0	0.3	1.4	0.5	0.9	---	---
Mites	30.6	1.4	21.6	4.2	29.4	0.8	1.9	1.9
Nematoda	---	---	---	---	---	---	---	---
Oligochaeta	---	---	1.2	4.2	12.7	78.5	53.3	53.3
Opiliones	---	---	---	---	3.2	1.5	---	---
Orthoptera	---	---	---	---	---	---	---	---
Plecoptera	---	---	---	---	---	---	---	---
Protura	7.8	0.1	---	---	---	---	---	---
Pseudoscorpionida	2.4	0.3	0.8	0.4	0.9	< 0.1	---	---
Psocoptera	---	---	---	---	---	---	---	---
Thysanoptera	---	---	---	---	1.4	0.1	---	---
Trichoptera	---	---	---	---	0.5	< 0.1	---	---
Unaccounted	0.4	1.2	0.2	< 0.1	---	---	0.1	0.1
No. of Salamanders	31		30		30		32	

	July - August							
	- Earthworm				+ Earthworm			
Site	1		2		3		4	
Taxon	Rel. Abun. (%)	Rel. Vol. (%)	Rel. Abun. (%)	Rel. Vol. (%)	Rel. Abun. (%)	Rel. Vol. (%)	Rel. Abun. (%)	Rel. Vol. (%)
Araneae	2.8	3.2	0.4	0.1	3.2	11.4	1.5	1.6
Archaeognatha	---	---	---	---	---	---	---	---
Cent/Mill	1.7	1.5	10.7	14.1	4.8	2.0	0.6	0.2
Coleoptera	17.8	54.3	6.3	45.8	5.9	19.0	4.7	7.1
Collembola	10.8	1.3	8.5	1.8	22.6	5.9	16.3	2.4
Diplura	---	---	---	---	0.5	< 0.1	---	---
Diptera	18.8	23.3	5.2	8.1	14.0	3.1	11.6	4.3
Gastropoda (Slugs)	4.5	4.3	---	---	1.1	9.2	0.3	2.6
Gastropoda (Snails)	---	---	2.2	1.3	1.1	0.3	3.5	0.3
Hemiptera	0.7	2.9	---	---	1.1	0.3	---	---
Hymenoptera	4.5	5.3	9.3	20.0	5.9	0.8	4.4	0.7
Isopoda	---	---	1.1	1.1	11.3	12.5	24.4	16.8
Lepidoptera	0.3	0.3	0.4	0.2	0.5	4.5	0.9	1.9
Mites	36.2	2.1	54.1	7.1	23.1	1.5	26.5	1.1
Nematoda	---	---	---	---	---	---	0.3	< 0.1
Oligochaeta	---	---	0.4	< 0.1	2.7	16.0	3.5	60.7
Opiliones	---	---	---	---	1.1	5.2	1.2	0.2
Orthoptera	---	---	---	---	---	---	0.3	0.1
Plecoptera	0.3	0.3	---	---	---	---	---	---
Protura	---	---	---	---	---	---	---	---
Pseudoscorpionida	0.7	< 0.1	0.4	0.2	---	---	0.3	0.1
Psocoptera	0.3	0.1	---	---	---	---	---	---
Thysanoptera	---	---	---	---	---	---	---	---
Trichoptera	---	---	---	---	---	---	---	---
Unaccounted	0.3	1.0	1.1	0.2	1.1	8.3	---	---
No. of Salamanders	30		35		35		31	

September - October								
	- Earthworm				+ Earthworm			
Site	1		2		3		4	
Taxon	Rel. Abun. (%)	Rel. Vol. (%)	Rel. Abun. (%)	Rel. Vol. (%)	Rel. Abun. (%)	Rel. Vol. (%)	Rel. Abun. (%)	Rel. Vol. (%)
Araneae	1.3	0.3	3.2	1.3	5.2	1.3	0.8	0.1
Archaeognatha	---	---	---	---	---	---	0.4	< 0.1
Cent/Mill	1.3	0.7	9.6	16.7	3.3	0.7	1.2	0.7
Coleoptera	30.9	63.9	5.0	22.7	3.3	0.5	3.2	0.4
Collembola	13.6	1.2	10.7	2.3	19.2	0.5	15.4	0.8
Diplura	---	---	---	---	---	---	---	---
Diptera	18.9	13.4	16.1	38.8	12.7	5.0	9.5	8.5
Gastropoda (Slugs)	2.4	0.1	0.7	6.9	1.4	2.0	0.8	2.3
Gastropoda (Snails)	---	---	1.4	0.4	1.4	0.2	7.1	0.5
Hemiptera	0.2	0.1	0.4	0.3	---	---	0.8	0.4
Hymenoptera	2.4	1.0	8.9	6.6	15.5	1.0	11.5	1.0
Isopoda	---	---	---	---	2.3	0.4	11.1	5.4
Lepidoptera	1.5	16.7	0.4	< 0.1	0.5	0.1	7.5	22.8
Mites	24.1	1.1	39.3	2.1	21.6	0.3	20.6	0.9
Nematoda	---	---	---	---	---	---	---	---
Oligochaeta	---	---	0.7	< 0.1	11.3	87.1	4.0	50.6
Opiliones	---	---	0.7	0.7	0.9	0.2	2.8	1.0
Orthoptera	---	---	---	---	---	---	---	---
Plecoptera	---	---	1.4	0.9	---	---	---	---
Protura	---	---	---	---	---	---	---	---
Pseudoscorpionida	0.7	0.1	---	---	---	---	---	---
Psocoptera	2.6	1.5	0.4	0.2	---	---	---	---
Thysanoptera	---	---	---	---	---	---	---	---
Trichoptera	---	---	---	---	---	---	---	---
Unaccounted	---	---	1.1	0.1	1.4	0.7	3.6	4.6
No. of Salamanders	32		33		32		31	
